# PReS-FINAL-2024: Responsiveness of the juvenile arthritis foot disability index

**DOI:** 10.1186/1546-0096-11-S2-P37

**Published:** 2013-12-05

**Authors:** AC Esbjörnsson, MD Iversen, EW Broström, S Hagelberg, M André

**Affiliations:** 1 Karolinska Institutet, Stockholm, Sweden; 2 North Eastern University, Boston, USA

## Introduction

Foot involvement is commonly described in children with JIA and management of foot disability is necessary to maximize physical function in children. The Juvenile Arthritis Foot disability Index (JAFI) is a questionnaire for assessing self-reported foot-related disability in children and adolescences with arthritis. The JAFI is a valid and reliable survey which is divided into three dimensions; impairment, activity limitations and participation restrictions (André 2004). However, its ability to measure change in foot related disability has not been established.

## Objectives

(a) To evaluate the responsiveness of the JAFI among a cohort of children with JIA undergoing treatment with Intra-articular corticosteroid injections (ICI). (b) To determine the association between JAFI change scores and the Child Health Assessment Questionnaire lower extremity dimension (CHAQ-low) change scores. We hypothesized that (a) the change in JAFI and CHAQ-low scores at 3 months following treatment from a group without foot impairments would demonstrate greater standardized response means (SRM = mean change/ SD change scores) and effect sizes (ES = mean change/ SD baseline scores) compared to scores from children with persistent foot disability. We also hypothesized that (b) JAFI change scores would moderately correlate (r = 0.4) with CHAQ-low change scores.

## Methods

35 children with JIA were consecutively recruited from a large tertiary care medical center pediatric rheumatology clinic. Twenty-eight participants were female; mean age (SD) 11(4) yrs. and mean (SD) disease duration of 4(4) yrs. 66% were diagnosed with polyarthritis. All children had active lower extremity inflammation and were scheduled for ICI in one or both feet. The children or parents (if child <10 yrs.) answered the JAFI and the CHAQ prior to injections, 3 months and 6 months after ICI. Scores from the CHAQ walking, rising, reaching and activity scales were summarized and divided into quartiles (CHAQ-low). To determine the responsiveness of the JAFI, children were separated into 2 groups based on standardized clinical examination, those without foot impairments at 3 months (n = 13) and those with foot impairments (n = 22) and standardized response means and effect sizes were calculated. Spearman correlations were performed to determine the association between JAFI dimensions and CHAQ- low change scores.

## Results

Both the SRM and the ES scores of the JAFI dimensions were higher for the group without foot impairments after 3 month (SRM; 0.59-1.00, ES; 0.52-0.92) as compared to the group with persistent foot impairments (SRM; -0.10-0.39, ES; -0.01-0.29). The JAFI impairment dimension showed the highest SRM and ES scores after ICI while the JAFI participation dimension showed the lowest scores. Correlations (r_s_) between JAFI dimensions and CHAQ-low change scores between baseline and 3 month were 0.44-0.67 and between baseline and 6 month were 0.46-0.48.

## Conclusion

In this study the JAFI appears to be a useful questionnaire for evaluating change in self-reported foot disability after treatment with ICI in children with JIA.

## Disclosure of interest

None declared.

